# Antibiotic Resistance of Enteric Bacteria in HIV-Infected Patients at the Banka Ad-Lucem Hospital, West Region of Cameroon

**DOI:** 10.1155/2019/9381836

**Published:** 2019-09-02

**Authors:** Ornella J. T. Ngalani, Armelle T. Mbaveng, Wiliane J. T. Marbou, Roland Y. Ngai, Victor Kuete

**Affiliations:** Department of Biochemistry, Faculty of Science, University of Dschang, Dschang, Cameroon

## Abstract

Human immunodeficiency virus (HIV) infection is a serious problem throughout the world and especially in developing countries. This study was conducted to define the bacterial aetiologies of enteric disorders and their association with CD4+ T-lymphocyte cell count and serum hs-CRP in HIV-seropositive patients coming for consultation at the AD-Lucem Banka Hospital. Stool samples from one-hundred HIV-positive patients with enteric disorders and forty HIV negative patients with enteric disorders were examined for the presence of bacteria by different diagnostic techniques. CD4+ T-lymphocyte count and serum hs-CRP of HIV-positive and HIV-negative patients were examined, respectively, by flux cytometry and the ELISA solid-phase direct sandwich method. Among all the participants, 39 (26.35%) were males and 109 (73.65%) were females. HIV-seropositive mean age (43.79 years) was significantly higher compared to HIV-seronegative (27.13 years) patients (*p* < 0.000). The average values of CD4+ T-cell count (*p* < 0.0001), lymphocytes (*p*=0.0258), monocytes (*p*=0.0317), and total WBC count (*p*=0.0277) were significantly higher in HIV− patients compared to HIV+ patients. *Salmonella* sp., *Escherichia coli*, and *Klebsiella pneumoniae* were more isolated in HIV+ patients 5 (83.33), 18 (75.00), and 37 (71.15) compared to HIV− patients 1 (16.67), 6 (25.00), and 15 (28.85), respectively. Majority of isolates were susceptible to IPM, NOR, and CIP. *Klebsiella pneumoniae*, the most prevalent isolate, showed resistance to AMC (45.95) in HIV+ patients, whereas in HIV− patients, *Enterobacter aerogenes* and *Shigella* sp. showed resistance to AMC (80.00% and 85.71%, respectively) and to CFM (80.00% and 57.14%, respectively). *Enterobacter aerogenes* (40.00%) and *Shigella* sp. (14.29) isolates showed multidrug resistance in HIV− patients, whereas *Escherichia coli* (5.56%) and *Klebsiella pneumoniae* (2.70%) showed multidrug resistance in HIV+ patients. Understanding the burden of bacteria disease in HIV patients as shown in the present study is important for planning effective control programs for the overall reduction of bacteria diseases in HIV-infected patients.

## 1. Introduction

More than 90% of microbial infections occur in less favoured areas of the planet every year [1]. They are the second leading cause of death in the world, with more than 17 million deaths each year, nearly 90% of them in developing countries [2]. Most of these infections are caused by bacteria that cause many diseases and, as a result, high rates of death. They constitute a major public health problem that affects all age groups and particularly immunocompromised individuals [3]. The bacterial opportunistic infections encountered in the latter have a variable severity. However, in the course of infection, the weakening of the immune system and increased vulnerability to opportunistic infections can occur [4], very often imposing medication abuse. Despite efforts to limit the transmission of enteric pathogens, the rate of resistance of various infectious agents varies across geographical areas and countries [5]. In Cameroon, the study conducted in Mbouda-Cameroun by Marbou and Kuete [6] showed a high prevalence of resistance of *Klebsiella* isolates to gentamycin, ceftriaxone, ciprofloxacin, doxycycline, and chloramphenicol in patients with HIV infection. Progressive decline in the immunological responses in HIV-infected patients makes them extremely susceptible to a variety of common and opportunistic infections. The risk of HIV- related illnesses is high when the CD4+ T-lymphocyte cell count is lower in infected patients. To better understand the bacterial aetiologies of enteric disorders in people living with HIV/AIDS in Bafang-Cameroon, this study was conducted to determine the association between CD4+ T-lymphocyte cell count, serum hs-CRP levels, and intestinal bacteria colonisation and their resistance in HIV-seropositive patients on consultation at the AD-Lucem Banka Hospital.

## 2. Materials and Methods

### 2.1. Study Design

This was a cross-sectional study carried out from July 2014 to May 2015 at the AD-Lucem Banka Hospital located at Bafang in Cameroon. It is one of a reference centre in Haut-Nkam Division of the west region in Cameroon.

### 2.2. Study Population

This study included the HIV/AIDS patients and HIV-seronegative patients on consultation for enteric disorders. One-hundred forty-eight (148) individuals were ready for voluntary participation in this study. They consisted of 100 seropositive individuals and 48 seronegative individuals. Patients, whose physicians prescribed stool examinations and have not received any specific antibacterial therapy in the previous two weeks, were included in this study. Not included in this study were pregnant women, diabetic patients, victims of burns, patients receiving oestrogens, and smokers.

### 2.3. Ethical Approval

Verbal and written consent were taken from all the study population. Patient's name/code, age, and sex were recorded. Ethical clearance was obtained from the Ethics Review and Consultancy Committee, Cameroon Bioethics Initiative (CAMBIN) Ref CBI/423/ERCC/CAMBIN.

### 2.4. HIV Testing

Screening for HIV sero-status was performed using OraQuick HIV (Ora Sure Technology, USA) test kits, as described by the manufacturer and as previously reported [7].

### 2.5. Blood and Stool Sample Collections

The stool samples were collected in sterile containers by taking all precautions to avoid contamination, while the blood samples were collected in two tubes, one containing anticoagulant (EDTA) and another was without anticoagulant.

### 2.6. Measurement of CD4+ T-Cell and hs-CRP

The CD4 lymphocytes were measured by cytometry technique using a flux cytometry (Apogee Flow systems Limited® Apogee Flow Systems, Hertfordshire, United Kingdom). High sensitivity C-reactive protein (hs-CRP) was measured using the ELISA solid-phase direct sandwich method (Sigma-Aldrich, St. Louis, USA) with ELx808™ Microplate reader (BioTek Instruments, Winooski, USA).

### 2.7. White Blood Cell (WBC) Count

Blood collected in EDTA tubes were gently agitated to avoid the formation of clots. Each sample was then introduced into a cellular counter (Mindray PE 6800®, PROCAN, China, Mainland). The automat calculated and automatically reported white blood cell, lymphocyte, monocyte, and granulocyte counts.

### 2.8. Bacterial Isolation and Identification

A single stool sample was collected from each study participant using a sterile and disinfectant-free container. Stool specimens were immediately taken to the laboratory and cultured within 30 minutes of collection on Salmonelle-Shigelle (SS) agar, Hektoen enteric agar, eosin methylene blue (EMB) agar, and MacConkey agar. The plates were incubated at 37°C for 24 h, and the isolated organisms were identified based on colonial morphology and Gram stain, and using API 20E galleries (Biomérieux, Lyon, France) as described by the manufacturer.

### 2.9. Antibiotic Sensitivity Test

Antibiotic susceptibility tests on the isolates were done by the Kirby–Bauer method [8], and the antibiotics tested included amikacin (AMI, 30 *μ*g), imipenem (IMP, 10 *μ*g), amoxicillin/clavuranic acid (AMC, 20/10 *μ*g), ciprofloxacin (CIP, 5 *μ*g), norfloxacin (NOR, 10 *μ*g), cefixime (CFM, 5 *μ*g), ceftriaxon (CRO, 30 *μ*g), and chloramphenicol (CHL, 30 *μ*g). Briefly, the test isolate was emulsified in peptone until the turbidity was similar to that of 0.5% McFarland's standard. A sterile cotton swab was dipped into the suspension and swabbed evenly across the entire surface of the agar plate in order to obtain a semiconfluent growth. After incubation, the zones of inhibition around the antibiotic discs were measured and interpreted based on the breakpoint criteria of the Clinical and Laboratory Standards Institute (CLSI) [9]. A quality control of antibiotic discs (Oxoid, UK), media (Accumix, Mol, Belgium), and incubation conditions was ensured using *Escherichia coli* ATCC 25922. Isolates showing resistance to three or more categories of antibiotics were considered as multidrug-resistant bacteria [10].

### 2.10. Statistical Analyses

All data were stored in a common database and statistically analysed by using Epi Info™ version 7.2.2.6 (CDC, 1600 Clifton Road Atlanta, GA 30329-4027 USA). The significant differences between HIV+ and HIV− were calculated by chi^2^ for categorical variables. To study the relation between the isolation rate of bacterial agents, HIV+ status, CD4 count, and hs-CRP, chi-square test and the Fisher's exact test were used. A *p* value of *p* < 0.05 was considered statistically significant.

## 3. Results

Out of the one-hundred forty-eight HIV+ and HIV− patients with enteric disorders recruited, 39 (26.35%) were males and 109 (73.65%) were females. Among males, 22 (56.41%) were HIV+ and 17 (43.59) were HIV−. Among females, 78 (71.56) were HIV+ and 31 (28.44) were HIV−. There was a significant difference in distribution of patients in different age groups. The maximum number of patients tested seropositive in the age group of [31–40] years (100.00%), followed by 91.11% and 88.00% in the age groups of >50 years and [41–50] years, respectively. The mean age of the participants in our study was significantly higher in HIV+ (43.79 ± 15.24 years) patients compared to HIV− (27.13 ± 13.09 years) patients, and the age ranged between 3 and 81 years (Table 1).

Table 2 shows the white blood cell count results, CD4+ T-cell count, and hs-CRP of HIV+ and HIV− patients with enteric disorders. It appears that the average values of CD4+ T-cell count (*p* < 0.0001), lymphocytes (*p*=0.0258), monocytes (*p*=0.0317), and total WBC count (*p*=0.0277) were significantly higher in HIV− patients compared to HIV+ patients.

**Figure 1:**
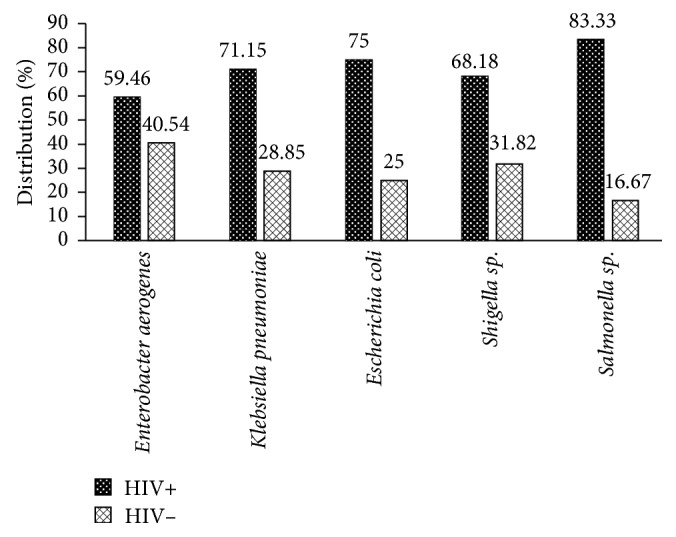
Distribution of intestinal bacteria pathogens among patients with enteric disorders enrolled in the study.

In the present study, bacteria were isolated from 97 (97.00%) HIV+ patients and 44 (91.67%) of HIV− patients. *Salmonella* sp., *Escherichia coli*, and *Klebsiella pneumoniae* were more isolated in HIV+ patients 5 (83.33), 18 (75.00), and 37 (71.15) compared to HIV− patients 1 (16.67), 6 (25.00), and 15 (28.85), respectively (Figure 1).

Table 3 shows the isolation of bacteria and their association with CD4+ T-lymphocyte counts and serum hs-CRP levels. Isolation of bacteria was more in patients who had CD4+ T-lymphocyte counts between 200 and 499 cells/*μ*l as compared to HIV-positive patients with CD4+ T-lymphocyte counts ≥500 cells/*μ*l. Isolation of *Klebsiella pneumoniae* was higher (54.05%) in patients with CD4+ T-lymphocyte counts 200–499 cells/*μ*l with statistically no significance. Concerning serum hs-CRP levels, isolation of bacteria was more in patients who had serum hs-CRP levels between 0 and 2.9 mg/l.

In our study, a majority of the isolates were susceptible to IPM, NOR, and CIP. *Klebsiella pneumoniae*, the most prevalent isolate, showed resistance to AMC (45.95) in HIV+ patients, whereas in HIV− patients, *Enterobacter aerogenes* and *Shigella* sp. showed resistance to AMC (80.00%, 85.71%), respectively, and to CFM (80.00%, 57.14%), respectively (Table 4).

**Figure 2:**
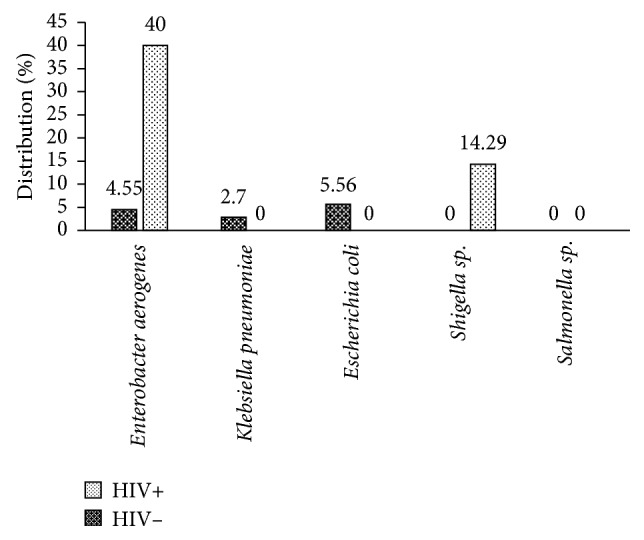
Frequency of occurrence of multidrug resistant (MDR) bacteria isolated from HIV+ and HIV− patients.

*Enterobacter aerogenes* (40.00%) and *Shigella* sp. (14.29) isolates showed multidrug resistance in HIV− patients, whereas *Escherichia coli* (5.56%) and *Klebsiella pneumonia* (2.70%) showed multidrug resistance in HIV+ patients (Figure 2).

## 4. Discussion

Intestinal bacteria are common causes of public health problems in Cameroon. In HIV− patients, intestinal infections are the leading causes of morbidity and mortality in developing countries, but their association with CD4+ T-cell counts and hs-CRP has received only cursory attention.

In the present study, most of the HIV+patients were in the age group [31–40] years, >50 years, and [41–50] years with 100%, 91.11%, and 88.00% of the cases, respectively. This result is similar to that of Marbou and Kuete's statistics with an estimated adult (31–50 age group) HIV prevalence of 55.88% in 2017 [6]. The female preponderance observed in the present study might have been due to the fact that females are more biologically vulnerable to HIV infections [11]. The mean age of HIV positive patients was 43.79 ± 15.24 years. The higher mean age of clinical presentation of HIV-positive patients may be because in our country, HIV infection is suspected when some clinical signs and symptoms start appearing or when illness is quite advanced.

We observed significant decrease of CD4+ T-cell count (*p* < 0.0001), lymphocytes (*p*=0.0258), monocytes (*p*=0.0317), and total WBC count (*p*=0.0277) in HIV+ patients compared to HIV− patients. These cells of the immune system are involved in protecting the body against both infectious diseases and foreign invaders. Haematopoietic system disorders are common throughout the course of HIV infection. Reduction in the absolute number of CD4 T cells occurs as one of the earliest immunologic abnormalities of HIV infection, and it is the most important prognostic indicator for risk of developing opportunistic infections [12].

The aetiology of enteric disorders in HIV-positive patients is multifactorial. We examined the stool samples for enteric bacteria. In the present study, bacteria were isolated from 97 (97.00%) HIV+ patients and 44 (91.67%) of HIV− patients with enteric disorders. *Salmonella* sp., *Escherichia coli*, and *Klebsiella pneumoniae* were more isolated in HIV+ patients 5 (83.33), 18 (75.00), and 37 (71.15) compared to HIV− patients 1 (16.67), 6 (25.00), and 15 (28.85), respectively. These bacteria are among those causing various infections associated with HIV patients [13]. Our findings are in accordance with those of the study conducted by Meremo et al., in Tanzania, where *Salmonella* spp. (39.4%) were the common bacteria isolated in febrile HIV adult patients [14].

The isolation of bacteria and their association with CD4+ T-lymphocyte counts and serum hs-CRP levels was studied. CD4+ T-lymphocyte count is used for management of HIV-infected individuals. In the present study, isolation of bacteria was higher in patients with CD4+ T-lymphocyte count between 200 and 499 cells/*μ*l. Relationship between the CD4+ T-lymphocyte count and the presence of bacteria in HIV+ patients was not significant. HIV-induced changes in cytokine responses to bacteria may influence susceptibility to bacterial infections and the consequent inflammatory response [15]. C-reactive protein (CRP) is a blood test marker for inflammation in the body, produced in the liver. In HIV+ patients, isolation of bacteria was more in patients who had serum hs-CRP levels 0–2.9 mg/l. Lower CRP levels have been shown in HIV+ patients. Malfunctions created by HIV infection may affect CRP synthesis when a patient is infected with bacteria [16].

Results of antimicrobial susceptibility tests revealed that most, majority of isolates were susceptible to IPM, NOR, and CIP. Meremo et al. also demonstrated bacteria susceptibility to IMP in HIV patients [14]. *Klebsiella pneumoniae*, the most prevalent isolate, showed resistance to AMC (45.95) in HIV+ patients, whereas in HIV− patients, *Enterobacter aerogenes* and *Shigella* sp. showed resistance to AMC (80.00% and 85.71%), respectively, and to CFM (80.00%, 57.14%), respectively. Bacteria antibiotics resistance in HIV+ patients compared to HIV− patients was also studied by Ba et al. in Nigeria [17].

Multidrug resistance was studied, and it appears that *Enterobacter aerogenes* and *Shigella* sp. isolates showed multidrug resistance in HIV− patients, whereas *Escherichia coli* and *Klebsiella pneumoniae* showed multidrug resistance in HIV+ patients. In general, the low multidrug resistance observed among the isolates from HIV+ individuals as compared to isolates from HIV− individuals could be as a result of strict adherence of HIV+ patients to the drugs prescribed only by their physicians.

This study that helps to understand the burden of bacteria disease in HIV patients could be of interest to physicians and other health professionals. This study due to its cross-sectional nature does not allow deducing a cause-and-effect relationship between parameters and presumed etiologic factors.

## 5. Conclusion

This study documents the association between CD4+ T-lymphocyte cell count, serum hs-CRP levels, and intestinal bacteria colonisation and their resistance in HIV+ patients on consultation at the Banka AD-Lucem Hospital. We noticed significant decrease of CD4+ T-cell count, lymphocyte, monocytes, and total WBC count in HIV+ compared to HIV− patients. No significant association between CD4+ T-lymphocyte cell count, serum hs-CRP levels, and intestinal bacteria colonisation was observed. IPM, NOR, and CIP were the most active antibiotics on pathogenic bacteria. *Enterobacter aerogenes* and *Shigella* sp. isolates showed multidrug resistance in HIV− patients, whereas *Escherichia coli* and *Klebsiella pneumoniae* showed multidrug resistance in HIV+ patients. Understanding the burden of bacteria disease in HIV patients and the variation by region is important for planning effective control programs for the overall reduction of bacteria diseases in HIV-infected patients.

## Figures and Tables

**Table 1:** Demographic profile of patients with enteric disorders enrolled in the study.

Parameters		HIV+ with enteric disorders, *N* = 100 (%)	HIV− with enteric disorders, *N* = 48 (%)	*p* value
Sex				0.0828
	Male (*N* = 39)	22 (56.41)	17 (43.59)	
	Female (*N* = 109)	78 (71.56)	31 (28.44)	

Age groups (years)				<0.0001
	[0–10]; (*N* = 5)	3 (60.00)	2 (40.00)	
	[11–20]; (*N* = 10)	5 (50.00)	5 (50.00)	
	[21–30]; (*N* = 43)	9 (20.93)	34 (79.07)	
	[31–40]; (*N* = 20)	20 (100.00)	0 (00.00)	
	[41–50]; (*N* = 25)	22 (88.00)	3 (12.00)	
	>50; (*N* = 45)	41 (91.11)	4 (8.89)	

Mean age; mean ± SD [min–max]	Total	43.79 ± 15.24 [3–81]	27.13 ± 13.09 [3–65]	<0.0001
	Male	46.45 ± 13.74 [15–61]	29.47 ± 14.17 [8–64]	0.0006
	Female	43.04 ± 15.64 [3–81]	25.84 ± 12.52 [3–65]	<0.0001

**Table 2:** White blood cell count results, CD4+ T-cell count, and hs-CRP of HIV-positive and HIV-negative patients with enteric disorders.

Parameter	HIV+ with enteric disorders (*n* = 100)		HIV− with enteric disorders (*n* = 48)		*p* value (between HIV+ and HIV−)
	Mean value ± SD	Range (min–max)	Mean value ± SD	Range (min–max)	
Total WBC (x 10^9^/L)	4.17 ± 1.28	2.00–8.30	5.13 ± 2.81	2.60–18.00	0.0277
Lymphocytes	1.91 ± 0.75	0.60–5.10	2.21 ± 0.76	1.10–4.00	0.0258
Granulocytes	1.80 ± 0.82	0.60–5.40	2.30 ± 2.15	0.70–10.90	0.1242
Monocytes	0.46 ± 0.20	0.20–1.30	0.61 ± 0.47	0.30–3.10	0.0317
CD4+ T cell	459.62 ± 166.80	63–958	951.94 ± 142.66	699–1365	<0.0001
hs-CRP	7.53 ± 10.88	0.11–50.40	9.81 ± 13.70	0.01–60.25	0.3154

**Table 3:** Bacteria and their association with CD4+ T-cell counts and hs-CRP in HIV-positive patients (*N* = 100).

Bacterial species	Total	CD4+ T cell counts				Hs-CRP (mg/l)			*p* value
		CD4 <200/*μ*l (*N*)	CD4 = 200–500/*μ*l (*N*)	CD4 >500/*μ*l (*N*)	*p* value	0–2.9 (*N*)	3–10 (*N*)	>10 (*N*)	
		*N* (%)	*N* (%)	*N* (%)		*N* (%)	*N* (%)	*N* (%)	
*Enterobacter aerogenes*	22	1 (4.55)	11 (50.00)	10 (45.45)	0.9325	7 (31.82)	10 (45.45)	5 (22.73)	0.5270
*Klebsiella pneumoniae*	37	0 (0.00)	20 (54.05)	17 (45.95)	0.2053	19 (51.35)	11 (29.73)	1 (18.92)	0.3357
*Escherichia coli*	18	1 (5.56)	11 (61.11)	6 (33.33)	0.7123	7 (38.89)	6 (33.33)	5 (27.78)	0.7369
*Shigella* sp.	15	2 (13.33)	8 (53.33)	5 (33.33)	0.2507	6 (40.00)	7 (46.67)	2 (13.33)	0.6199
*Salmonella* sp.	5	0 (0.00)	3 (60.00)	2 (40.00)	0.8535	3 (60.00)	0 (0.00)	2 (40.00)	0.1061

**Table 4:** Antibiotic resistance profile of bacterial isolates from HIV-seropositive and HIV-seronegative patients.

Antibiotics		*Enterobacter aerogenes*		*Klebsiella pneumoniae*		*Escherichia coli*		*Shigella* sp.		*Salmonella* sp.	
		HIV+ *n* (%)	HIV− *n* (%)	HIV+ *n* (%)	HIV− *n* (%)	HIV+ *n* (%)	HIV− *n* (%)	HIV+ *n* (%)	HIV− *n* (%)	HIV+ *n* (%)	HIV− *n* (%)
IPM	R	0 (0.00)	0 (0.00)	0 (0.00)	0 (0.00)	0 (0.00)	0 (0.00)	0 (0.00)	0 (0.00)	0 (0.00)	0 (0.00)
	I	0 (0.00)	0 (0.00)	0 (0.00)	0 (0.00)	0 (0.00)	0 (0.00)	0 (0.00)	0 (0.00)	0 (0.00)	0 (0.00)
	S	22 (100)	15 (100.00)	37 (100)	15 (100.00)	18 (100)	6 (100.00)	15 (100)	7 (100.00)	5 (100)	1 (100.00)

AMC	R	13 (59.09)	12 (80.00)	17 (45.95)	6 (40.00)	6 (33.33)	3 (50.00)	5 (33.33)	6 (85.71)	2 (40.00)	0 (0.00)
	I	6 (27.27)	1 (6.67)	9 (24.32)	5 (33.33)	8 (44.44)	2 (33.33)	4 (26.67)	0 (0.00)	2 (40.00)	1 (100.00)
	S	3 (13.64)	2 (13.33)	11 (29.73)	4 (26.67)	4 (22.22)	1 (16.67)	6 (40.00)	1 (14.29)	1 (20.00)	0 (0.00)

NOR	R	0 (0.00)	1 (6.67)	0 (0.00)	0 (0.00)	0 (0.00)	0 (0.00)	0 (0.00)	1 (14.29)	0 (0.00)	0 (0.00)
	I	2 (9.09)	1 (6.67)	0 (0.00)	0 (0.00)	0 (0.00)	0 (0.000	0 (0.00)	1 (14.29)	0 (0.00)	0 (0.00)
	S	20 (90.91)	13 (86.67)	37 (100.00)	15 (100.00)	18 (100.00)	6 (100.000	15 (100.00)	5 (71.43)	5 (100.00)	1 (100.00)

CIP	R	1 (4.55)	0 (0.00)	0 (0.00)	0 (0.00)	0 (0.00)	0 (0.00)	0 (0.00)	0 (0.00)	0 (0.00)	0 (0.00)
	I	2 (9.09)	0 (0.00)	1 (2.70)	0 (0.00)	0 (0.00)	0 (0.00)	0 (0.00)	0 (0.00)	0 (0.00)	0 (0.00)
	S	19 (86.36)	15 (100.00)	36 (97.30)	15 (100.00)	18 (100.00)	6 (100.00)	15 (100.00)	7 (100.00)	5 (100.00)	1 (100.00)

CFM	R	4 (18.18)	12 (80.00)	10 (27.03)	2 (13.33)	6 (33.33)	1 (16.67)	2 (13.33)	4 (57.14)	1 (20.00)	1 (100.00)
	I	1 (4.55)	1 (6.67)	4 (10.81)	1 (6.67)	1 (5.56)	1 (16.67)	3 (20.00)	1 (14.29)	2 (40.00)	0 (0.00)
	S	17 (77.27)	2 (13.33)	23 (62.16)	12 (80.00)	11 (61.11)	4 (66.67)	10 (66.67)	2 (28.57	2 (40.00)	0 (0.00)

CRO	R	4 (18.18)	10 (66.67)	10 (27.03)	4 (26.67)	4 (22.22)	2 (33.33)	3 (20.00)	4 (57.14)	1 (20.00)	0 (0.00)
	I	9 (40.91)	1 (6.67)	12 (32.43)	5 (33.33)	4 (22.22)	0 (0.00)	4 (26.67)	2 (28.57)	1 (20.00)	0 (0.00)
	S	9 (40.91)	4 (26.67)	15 (40.54)	6 (40.00)	10 (55.56)	4 (66.67)	8 (53.33)	1 (14.29)	3 (60.00)	1 (100.00)

AMK	R	2 (9.09)	0 (0.00)	0 (0.00)	0 (0.00)	0 (0.00)	0 (0.00)	0 (0.00)	0 (0.00)	0 (0.00)	0 (0.00)
	I	8 (36.36)	8 (53.33)	18 (48.65)	12 (80.00)	8 (44.44)	3 (50.00)	10 (66.67)	4 (57.14)	4 (80.00)	0 (0.00)
	S	12 (54.55)	7 (46.67)	19 (51.35)	3 (20.00)	10 (55.56)	3 (50.00)	5 (33.33)	3 (42.86)	1 (20.00)	1 (100.00)

CHL	R	5 (22.73)	8 (53.33)	2 (5.41)	1 (6.67)	3 (16.67)	0 (0.00)	0 (0.00)	2 (28.57)	1 (20.00)	0 (0.00)
	I	2 (9.09)	1 (6.67)	4 (10.81)	3 (20.00)	0 (0.00)	0 (0.00)	4 (26.67)	2 (28.57)	2 (40.00)	1 (100.00)
	S	15 (68.18)	6 (40.00)	31 (83.78)	11 (73.33)	15 (83.33)	6 (100.000)	11 (73.33)	3 (42.86)	2 (40.00)	0 (0.00)

Imipeneme, IMP; amoxicillin/clavuranic acid, AMC; norfloxacin, NOR, ciprofloxacin, CIP; cefixime, CFM; ceftriaxon, CRO; amikacin, AMK, chloramphenicol, CHL.

## Data Availability

The data used to support the findings of this study are included within the supplementary information file.

## Abbreviations

AMC: Amoxicillin/clavuranic acid
AMI: Amikacin
CFM: Cefixime
Chapman: Mannitol salt agar
CHL: Chloramphenicol
CIP: Ciprofloxacin
CLSI: Clinical and Laboratory Standards Institute
CRO: Ceftriaxon
CRP: C-reactive protein
ELISA: Enzyme-linked immunosorbent assay
EMB: Eosin methylene blue
HIV: Human immunodeficiency virus
IMP: Imipenem
MDR: Multidrug resistance
MHA: Muller Hilton agar
NOR: Norfloxacin
SS: Salmonelle-Shigelle agar
WBC: White blood cells.

## Supplementary Materials

Supplementary Materials Supporting information supporting file (XLS): all raw data generated to support the findings of this study.
